# Role of the Interaction of Tumor Necrosis Factor-α and Tumor Necrosis Factor Receptors 1 and 2 in Bone-Related Cells

**DOI:** 10.3390/ijms23031481

**Published:** 2022-01-27

**Authors:** Hideki Kitaura, Aseel Marahleh, Fumitoshi Ohori, Takahiro Noguchi, Yasuhiko Nara, Adya Pramusita, Ria Kinjo, Jinghan Ma, Kayoko Kanou, Itaru Mizoguchi

**Affiliations:** Division of Orthodontics and Dentofacial Orthopedics, Tohoku University Graduate School of Dentistry, 4-1 Seiryo-machi, Aoba-ku, Sendai 980-8575, Japan; marahleh.aseel.mahmoud.t6@alumni.tohoku.ac.jp (A.M.); fumitoshi.ohori.t3@dc.tohoku.ac.jp (F.O.); takahiro.noguchi.r4@dc.tohoku.ac.jp (T.N.); yasuhiko.nara.q6@dc.tohoku.ac.jp (Y.N.); adya.pramusita.q6@dc.tohoku.ac.jp (A.P.); ria.kinjou.p5@dc.tohoku.ac.jp (R.K.); ma.jinghan.s1@dc.tohoku.ac.jp (J.M.); kanou.kayoko.s7@dc.tohoku.ac.jp (K.K.); mizo@tohoku.ac.jp (I.M.)

**Keywords:** TNF-α, TNF receptor-1, TNF receptor-2, osteoclast, osteoblast, osteocyte

## Abstract

Tumor necrosis factor-α (TNF-α) is a pleiotropic cytokine expressed by macrophages, monocytes, and T cells, and its expression is triggered by the immune system in response to pathogens and their products, such as endotoxins. TNF-α plays an important role in host defense by inducing inflammatory reactions such as phagocytes and cytocidal systems activation. TNF-α also plays an important role in bone metabolism and is associated with inflammatory bone diseases. TNF-α binds to two cell surface receptors, the 55kDa TNF receptor-1 (TNFR1) and the 75kDa TNF receptor-2 (TNFR2). Bone is in a constant state of turnover; it is continuously degraded and built via the process of bone remodeling, which results from the regulated balance between bone-resorbing osteoclasts, bone-forming osteoblasts, and the mechanosensory cell type osteocytes. Precise interactions between these cells maintain skeletal homeostasis. Studies have shown that TNF-α affects bone-related cells via TNFRs. Signaling through either receptor results in different outcomes in different cell types as well as in the same cell type. This review summarizes and discusses current research on the TNF-α and TNFR interaction and its role in bone-related cells.

## 1. Introduction

Skeletal health, architecture, and homeostasis are functions of the constant process of bone remodeling [1]. Bone-resorbing osteoclasts, bone-forming osteoblasts, and the mechanosensory osteocytes cooperate to tightly regulate bone remodeling [2,3,4]. Tumor necrosis factor-α (TNF-α) exhibits important functions in host defense, initiates and propagates inflammatory cascades by signaling in various cell types, and activates cytocidal systems by mononuclear phagocytes [5].

Osteoclasts, descendants of hematopoietic stem cells, are responsible for bone resorption [6], and disproportionately increased osteoclast activity leads to excessive bone loss in different conditions, such as rheumatoid arthritis, periodontal diseases, and periprosthetic bone destruction [7,8,9]. Recruitment of osteoclasts induced by TNF-α is central to the inflammatory element of destructive bone diseases [10], and TNF-α-targeted drugs are a known effective tool for suppressing inflammatory bone destruction in diseases such as rheumatoid arthritis [11].

Osteoblasts are derived from mesenchymal stem cells [12]. Their activity is linked to osteoporosis, in which bone density is reduced because of a combination of increased resorption by osteoclasts and decreased expression of bone anabolic factors which influence osteoblast activity [13,14]. TNF-α has a dual effect in this respect: it induces osteoclastic bone destruction and it inhibits osteoblast differentiation [15].

Osteocytes are terminally differentiated osteoblasts entombed in a mineralized bone matrix [16,17]. They are connected to other cells and the bone surface via an extensive canalicular system [18], enabling osteocytes to sense mechanical stress finely, remodel the surrounding matrix, and regulate mineral metabolism [19]. Osteocytes produce various cytokines that signal in autocrine, paracrine, and endocrine modes [20]; of these cytokines is the receptor activator of nuclear factor κB ligand (RANKL), which is vital for osteoclast formation in the mature remodeling skeleton [21,22]. Osteocytes express TNFR1 and TNFR2, and TNF-α enhances RANKL expression in osteocytes directly and induces osteoclast formation both in vitro and in vivo [23].

TNF-α interacts with two receptors: the 55 kDa tumor necrosis factor receptor-1 (TNFR1), also called CD120a and p55, and the 75 kDa tumor necrosis factor receptor-2 (TNFR2), also called CD120b and p75 [24]. TNFR1 and TNFR2 are also stimulated by another cytokine, lymphotoxin alpha (LT-α, previously known as TNF-β). LT-α is closely related to TNF-α, and both are activated by similar stimuli [25]. TNFR1 and TNFR2 exist in two forms, on the plasma membrane, and in a soluble state after TACE activation. The intracellular domains of the receptors are unrelated, and these receptors mediate signaling in independent pathways. TNFR1 is a model death receptor primarily involved in cytotoxicity, and TNFR2 plays a role in proliferation [26,27].

This review outlines the current knowledge on the interaction of TNF-α with TNFR1 and TNFR2 in bone-related cells.

## 2. TNFR

### 2.1. TNFR1

The tumor necrosis factor receptor superfamily (TNFRSF) is characterized by the ability to bind tumor necrosis factor superfamily (TNFSF) ligands [25,28]. The TNFRSF comprises 29 receptors, including TNFR1 and TNFR2 and the TNFSF comprises 19 ligands [25,29]. TNFR1 is expressed on almost all cells [30] and has extracellular TNF-binding structures characterized by four repeated cysteine-rich domains (CRDs: CRD1, CRD2, CRD3, and CRD4) [31,32]. TNFR1 exists in its trimetric form before stimulation by TNF-α. Monomeric TNF receptors typically trimerize around TNF ligands that recruit TNF receptor-associated factors (TRAF) to the membrane and initiate a downstream signaling cascade [33,34]. TNFR1 has a cytoplasmic region, designated as the death domain (DD), which initiates a cytotoxic signal [35]. TNFR1 activation of NF-κB is dependent on several molecules such as the receptor-interacting protein kinase 1 (RIPK1, also known as RIP1), TNFR1-associated death domain (TRADD), TRAF2, and Fas-associated death domain (FADD) [36]. TNFR1 activates various responses. For example, TNFR1 promotes inflammatory responses, regulates cytokines, chemokines, adhesion molecules and other receptors, as well as induces the migration of neutrophils, and regulates the complement system [25]. The interaction of TNF-α and TNFRs is significantly involved in the development of lymphatic tissue; TNFR1-deficient mice show reduced numbers of Peyer’s patches and exhibit distressed germinal center formation in the white pulp of the spleen and follicular dendritic cell development and localization. TNFR1 is the likely principal receptor transducing TNF signals during lymphoid tissue development because TNFR2-deficient mice do not show changes in lymphatic organogenesis [37]. Studies have also shown that TNFR1 mediates the immune response necessary to combat infection by microbes such as mycobacteria [38] and Listeria monocytogenes [39], while TNFR2 absence has a negligible effect on supporting this response [40]. In addition, TNFR1 is the major receptor mediating host defense against viral infection [41].

### 2.2. TNFR2

The expression of TNFR2 is restricted compared to the ubiquitous expression of TNFR1. TNFR2 is expressed on endothelial cells, mesenchymal stem cells, and immune cells, such as macrophages, monocytes, T cells, B cells, and NK cells [42,43,44,45,46,47], and neural cells [48]. The extracellular region of TNFR2 contains CRDs (CRD1, CRD2, CRD3, and CRD4) as observed in TNFR1 [31,32], and TNFR2 exists in its trimetric form before stimulation by TNF-α. However, in contrast to TNFR1, TNFR2 lacks a death domain in the intracellular region of the receptor [49]. Although TNFR2 is primarily involved in cell proliferation, activation, and survival, it also induces apoptosis and enhances TNFR1-mediated cell death [50,51]. Upon activation of TNFR2, TRAF1 and TRAF2 interact with the TNFR2 cytoplasmic domain [34]. TNFR2’ signaling through TRAF2 induces JNK activation and uses ASK1 as a mediator to activate NF-κB and facilitate anti-apoptotic signals [52]. Several papers showed that TNFR1, not TNFR2, is the essential receptor for preventing bacterial infections [53,54,55], and TNFR2 has been shown to induce viral resistance in vivo [56]. TNFR1-deficient mice with polymicrobial septic shock attenuate the disease; however, TNFR2-deficient mice with polymicrobial septic shock experience worse symptoms and shorter survival [57]. Together, these studies indicate that TNFR 1 seems to mediate immune responses to bacterial and viral challenges, whereas TNFR2 may be primarily involved in the response to viral infections.

## 3. Osteoclasts

### 3.1. TNFR1 and TNFR2 in Osteoclasts and the Role of the Interaction of TNF-α and TNFRs in Osteoclast Formation

RANKL is an essential factor for osteoclast differentiation [58,59] and binds to the membrane-bound receptor RANK on the surface of osteoclast precursors. RANK expression is induced by the macrophage colony-stimulating factor (M-CSF) [60]. While RANKL is a requisite molecule for osteoclast formation, some researchers reported that osteoclast formation still proceeds without RANKL in the presence of TNF-α. For example, osteoclasts form from bone marrow macrophages in vitro independent of RANKL [61,62]. Other researchers argued that TNF-α amplifies the effect of RANKL but does not act alone because TNF-α induces osteoclast formation, given that low concentrations of RANKL were also supplied [63]. To clarify this issue, TNF transgenic mice were crossed with RANK-/- mice, and the lack of RANK resulted in severe osteopetrosis even though TNF-α was over-expressed [64]. Thus, TNF-α mediated osteoclast formation depends on RANKL and is considered to enhance RANKL signaling rather than act alone. Nonetheless, there are particular settings in which TNF-α can induce osteoclast formation independent of RANKL if sufficient cofactors are present. For example, factors such as M-CSF and transforming growth factor-β (TGF-β) act in conjunction with TNF-α to induce osteoclast differentiation in mouse models lacking RANKL, RANK, and TRAF6. Although the formed osteoclasts were not functional, osteoclast functionality was recovered by TRAF6 activation via IL-1 [65]. These findings suggest that TNF-α-induced osteoclast formation acts independent of RANK/RANKL, given that additional cofactors are supplemented and that TRAF6 is essential for osteoclast activation and function. Further studies using different mouse models and culture conditions are necessary to clarify the role of these molecules together and alone.

TNF-α plays a central role in inflammatory osteoclast formation and affects cells involved in osteoclast formation, including macrophages, stromal cells, and T cells expressing RANKL, which enables their osteoclastogenic potential [66,67]. Our previous studies showed that TNF-α induces osteoclast formation and bone resorption in vivo, possibly targeting those cells [68,69,70,71,72,73]. Thus, a better understanding of the contribution of TNF-α in those target cell types may help identify critical therapeutic strategies. A previous study reported that osteoclast precursors are direct targets of TNF-α in vitro [63]. To test the contribution of TNF-α to osteoclast formation, we created four kinds of chimeric mice by manipulating the bone marrow cell content. We transplanted wild-type marrow into lethally irradiated mice lacking TNFRI and TNFRII. The result was mice with stromal cells and macrophages bearing TNFRs, mice with only stromal cells bearing TNFRs, mice with only macrophages bearing TNFRs, and mice with stromal cells and macrophages deficient in TNFRs. Osteoclast formation was evaluated after injection of TNF-α into the supracalvariae of these chimeric mice. The results indicated that stromal cells contribute more to osteoclast formation than macrophages, although both macrophages and stromal cells were found to be direct targets of TNF-α [68,74]. TNF-α acts on macrophages to increase their RANK expression and on stromal cells to increase their expression of RANKL and M-CSF, the latter being essential in inducing RANK expression after monocyte differentiation into osteoclast precursors [68,74].

Several reports showed that the compression force in orthodontic tooth movement (OTM) in mice induces the expression of TNF-α [75,76,77,78,79,80]. Our studies found that TNF-α plays an essential role in OTM in both TNFR1- and TNFR2-deficient mice, which experienced less tooth movement distance compared to wild-type mice [81,82,83,84,85,86]. Furthermore, we showed that chimeras made of TNFRs deficient mice and wild-type mice can be used to examine the type of cell targeted by TNF-α for osteoclast formation during OTM. These findings suggested that the response of stromal cells to TNF-α is a crucial factor for osteoclast formation and bone resorption in OTM [83].

Analysis of bone marrow cells derived from mice differentially expressing TNFR1 or TNFR2 revealed that TNFR1 induced osteoclast formation results are comparable to those obtained in wild-type mice, and marrow from mice expressing only TNFR2 could not stimulate osteoclast formation [87]. Moreover, in the OTM setting, the number of osteoclasts in TNFR1-deficient mice was lower than that in wild-type mice [75]. In contrast, when tooth movement was applied to mice deficient in either TNFR1 or TNFR2, less tooth movement and less osteoclast formation were observed in TNFR2-deficient mice compared with those observed in TNFR1-deficient mice [81]. These results suggested that selective signaling through TNFR1 or TNFR2 has different outcomes and that TNFR1 plays an essential role in TNF-α-induced osteoclast formation.

### 3.2. TNF-α Signaling Pathways via TNFR1 and TNFR2 for Osteoclast Formation

Understanding TNFR-TNF-α signaling goes hand in hand with understanding the signaling pathways involved in osteoclast differentiation by M-CSF and RANKL. M-CSF is one of the early cytokines involved in the differentiation of monocytes to osteoclasts and the survival of osteoclasts. Osteoclast precursors express the M-CSF receptor c-Fms, and M-CSF, and in turn, activates the mitogen-activated protein kinase (MAPK) pathways, PI3K, and Akt [60].

Differentiation involves the expression of RANK on the surface of osteoclast precursors, which are activated by RANKL, leading to the induction of a plethora of effectors. The downstream signals include MAPK pathways, such as family-mediated JNK, p38, NF-κB, and the AP-1 family, such as c-Fos and TRAF [88]. NF-κB- and c-Fos-deficient mice showed osteopetrosis symptoms, suggesting that NF-κB and c-Fos are essential molecules for osteoclast formation [89,90,91]. TRAF6-deficient mice were used to examine the role of TRAF6 for RANKL-induced osteoclast formation; these mice exhibited reduced numbers of osteoclasts and osteopetrosis symptoms [92,93]. The transcription factors AP-1 and NF-κB promote osteoclast formation and activate the nuclear factor of activated T-cell c1 (NFATc1). NFATc1 is required for osteoclast formation as the master transcription factor in osteoclast formation [94]. NFATc1 is activated by calcineurin-mediated dephosphorylation in osteoclast formation [95]. NFATc1 migrates into the nucleus and binds to promoters of osteoclast-related genes such as TRAP, the calcitonin receptor, and cathepsin K genes [94]. The osteoclast-associated receptor (OSCAR) binds to adaptor molecules associated with immunoreceptor tyrosine-based activation motifs (ITAM) in osteoclast precursors and activates NFATc1 as co-stimulatory signals for RANKL [96].

Previous studies examined whether TRAF6 is required for TNF-α-induced osteoclast formation using osteoclast precursors from TRAF6-deficient mice. The results showed that TNF-α cannot induce osteoclast formation and suggested that TRAF6 was required for TNF-α-induced osteoclast formation in vitro [97]. TRAF6 is not a common adaptor protein for TNFRs. TNF-α synergistically enhances RANKL-induced osteoclast formation but does not act alone, because TNF-α can induce osteoclast formation with RANKL [63]. These studies suggest that TNF-α-induced osteoclast formation might be necessary for the existence of RANKL, and further research is required to explore this possibility. Another study analyzed the role of TRAF2 in TNF-α-induced osteoclast formation using fetal liver cells from TRAF2-deficient mice and found that osteoclast formation induced by TNF-α was severely inhibited. Fewer osteoclasts were formed via RANKL from progenitors from TRAF2-deficient mice than those from wild-type mice [98], indicating that TRAF2 signaling is essential for TNF-α-induced osteoclast formation. The results suggested that TRAF2 signaling promotes TRAF6 signaling in RANKL-induced osteoclast formation. Another study reported that TNF-α-induced osteoclast formation was attenuated in TRAF5-deficient mice in vitro [98]. Further studies are needed to elucidate which TRAF is essential for TNF-α-induced osteoclast formation.

TNF-α signaling induces different biological reactions depending on the receptor activated, either TNFR1 or TNFR2, because each receptor mediates distinct intracellular signals. A study in TNFR1- or TNFR2-deficient mice for TNF-α-induced osteoclast formation in vitro revealed that TNFR1 enhanced osteoclast formation and TNFR2 inhibited osteoclast formation [87]. TNF-α induces activation of MAPKs through phosphorylation of ERK, p38, and JNK in osteoclast precursors [69,85,99]. In addition, TNF-α induces phosphorylation of ATF2 downstream of MAPKs [99] and induces phosphorylation of IκB in osteoclast precursors [85,86,100].

TNF-α has been shown to support osteoclast survival [101] by activating Akt phosphorylation in osteoclasts. TNF-α-induced signaling is inhibited by the PI3K inhibitor LY294002 and the Src family kinase-selective inhibitor, PP1. These inhibitors decreased osteoclast survival. Furthermore, TNF-α-enhanced ERK phosphorylation was observed. In contrast, treatment with PD98059, which is a specific inhibitor of the ERK activating kinase MEK-1, resulted in the inhibition of TNF-α-induced phosphorylation of ERK and the survival of osteoclasts [101]. TNF-α acts mainly via NF-κB activation, leading to the transcription and production of anti-apoptotic factors [102]. TNF-α signaling converges on the mammalian target of rapamycin (mTOR) as part of the anti-apoptotic action of osteoclasts. The TNF-α signaling intermediates for mTOR/ribosomal protein S6K activation include PI3K, ERK, and Akt. Specific inhibitors of these factors inhibited TNF-α-activated S6K and enhanced osteoclast apoptosis. Furthermore, mTOR regulates protein translation through S6K, 4E-BP1, and S6, and the inhibition of these molecules affects protein translation and induces osteoclast apoptosis [103].

TNF-α was indicated to induce actin ring formation, which is a required osteoclast formation in vitro [104]. Previous studies reported that TNF-α regulated forming and a precursor zone for the matured sealing-ring and the nascent sealing zone by L-plastin (LPL) phosphorylation [105,106,107]. TNF-α stimulates the assembly of actin aggregates at the early stage of sealing-ring formation, independent of αvβ3 integrin signaling. TNF-α changed the expression and phosphorylation levels of LPL. In osteoclasts plated on dental bone slices and treated with TNF-α, changes in actin organization on the nascent sealing zone and sealing-ring formation are mutually related with cortactin proteins, and LPL phosphorylation was observed [108]. The MAPK pathway is one of the signaling pathways leading to LPL phosphorylation [109,110]. PKA and PKC induce phosphorylation of LPL in other cell types; however, both PKA and PKC were not observed as downstream of the TNFR1 interacted with TNF-α in osteoclasts [111]. Further studies examined the molecular mechanism of L-plastin phosphorylation and the subsequent formation of the nascent sealing zone in TNF-α-treated osteoclasts. The results showed that the TNF-α signaling pathway via TNFR1 compromised an Src-PI3K-TRAF-6-Rho/Rho-kinase axis for activating the phosphorylation of LPL and regulating nascent sealing zone formation [111].

The signaling pathways activated via TNFR1 and TNFR2 have been intensively studied in a variety of other cell types; few studies have examined TNF-α-signaling in osteoclast precursors/osteoclasts. Further studies are needed to elucidate the relationship between the signaling downstream of the interaction of TNF-α with TNFRs in osteoclast differentiation.

## 4. Osteoblasts

### 4.1. TNFR1 and TNFR2 in Osteoblasts

Osteoblasts differentiate from multipotent mesenchymal stem cells (MSCs) capable of producing adipocytes, chondrocytes, fibroblasts, and skeletal muscle cells [112,113,114,115,116]. MSCs commit to the osteoblastic pathway under the transcriptional control of Runx2 (also known as Cbfa1, AML-3, or Pepb2αA) via subnuclear localization and binding to its target DNA foci and the transcription factor osterix (Osx) acting downstream of Runx2 [117,118,119,120,121].

Reports on the expression of TNFRs by osteoblasts have yielded inconsistent results. While studies have agreed on the expression of TNFR1, TNFR2 has been considered by some authors as “not detected” in MG63 osteosarcoma cells and in non-transformed osteoblasts made from mesenchymal stem cells using unamplified gene screening [122]. While TNFR1 is generally expressed among all cell types (except erythrocytes), TNFR2 expression is restricted to immune cells, endothelial cells, and a few other cell types, including MSCs, which express an osteogenic proliferative potential [123,124]. In this review, we continue with the presumption that osteoblasts express both TNFRs because TNFR2-deficient osteoblasts exhibit a phenotype distinct from that of wild-type or TNFR1-deficient osteoblasts [125,126,127]. In the following sections, signaling in osteoblasts through TNFRs is discussed in the inhibition of osteoblast differentiation, apoptosis, and negatively influencing bone remodeling.

### 4.2. The Role of the Interaction of TNF-α and TNFRs in Osteoblast Differentiation

TNF-α inhibits osteoblast differentiation in cells from multiple cell sources: fetal calvarial cells, the MC3T3 pre-osteoblastic cell line, and murine marrow stromal cells [128,129]. Inhibition occurs in a temporal fashion at a critical time in cell culture (days 2–14 of a 21-day culture) and at very low doses [130]. TNFR-deficient mice exhibit an increased peak of basal bone mass and TNFR1-deficient mice display an increased basal bone mass in different anatomic positions and overall, because of increased bone formation [127].

TNFR1 is the receptor responsible for TNF-α-mediated osteoblast differentiation inhibition, while TNFR2 is dispensable in this process. While wild-type or TNFR2-deficient MSCs cultured with TNF-α exhibit the complete inhibition of osteoblast differentiation, nodular formation, and mineralization as well as decreased alkaline phosphatase activity and osteocalcin expression, TNFR1-deficient MSCs are completely unresponsive to TNF-α and exhibit no effects on osteoblast differentiation. Researchers reasoned that cytotoxicity might be why TNF-α inhibits osteoblast differentiation—because reducing the pool of precursor cells led to a decrease in the number of available osteoblasts. However, MC3T3 apoptotic cell death was not the reason why TNF-α blocks differentiation to osteoblasts, because treatment with caspase inhibitors did not reverse the inhibitory effects of TNF-α [125]. TNFR1 signaling is sufficient to inhibit osteoblast differentiation, which cannot be attributed to increased cell death. TNFR2, although dispensable, confers some protection against the actions of TNF-α [125,130]. TNFR2 did not affect osteoblast differentiation, and it decreased cell sensitivity to TNF-α when cells were treated with murine TNF-α; however, it showed no effect when cells were treated with human TNF-α. This is due to the ability of TNF-α to activate mouse TNFR1 and TNFR2 to similar degrees, whereas human TNF-α activates mouse TNFR1 but does not stimulate mouse TNFR2 [50].

The protective effect of TNFR2 on osteoblast differentiation is puzzling as TNFR2 has been proposed to assist TNFR1 signaling through ‘’ligand passing,’’ in which TNFR2 increases the concentration of TNF-α around TNFR1 due to the kinetic properties of TNF-α association and dissociation from TNFR2 [131]. Conversely, the protective effects of TNFR2 can be explained by the presence of a proteolytic cleavage site encoded by exon 6 in the TNFR2 gene. Cleavage at the extracellular domain of TNFR2 allows it to be released as an active soluble receptor (sTNFR2) that competes with TNFR1, antagonizing TNF-α effects [132]. Alternatively, other studies suggested that, at specific concentrations, TNFR2 may dampen the effect of TNF-α and alter the strength of signaling through TNFR1 in the same cell [133]. TNFR2 is strongly activated by mTNF-α, leading to cellular death, but is resistant to activation by sTNF-α; this observation, in addition to the kinetics of receptor binding, may explain the different response of osteoblasts harboring one of either receptor [134].

TNF-α inhibits osteoblast differentiation distal to the signaling of known osteogenic inductive proteins (IGF-I, BMP-2, BMP-6, and LMP-1) and by directly affecting Runx2 activity at multiple levels. First, TNF-α regulates Runx2 at the transcriptional level by decreasing mRNA expression of the two major Runx2 isoforms (P1/MASNS to 50%, P2/MRIPV to 90%), and second, by reducing mRNA half-life and stability, which translates into a reduction in the Runx2 nuclear content as well as a reduction in Runx2 binding to promoter regions [128,130]. Interestingly, TNF-α antiproliferative and apoptotic effects on osteoblasts may be facilitated by Runx2 itself; hMSCs transfected with siRNA designed to inhibit Runx2 expression are less sensitive than hMSCs transfected with a nonspecific siRNA. This may explain the conflicting results TNF-α has on osteoblast apoptosis (see below) as TNF-α effects on osteoblasts require active Runx2 expression [129].

Various post-transcriptional and post-translational mechanisms regulate Runx2 expression and activity. BMP-2 regulates Runx2 via stabilizing its binding to Smads and preventing its degradation by Smurf1/2 [135,136]. TNF-α upregulates Smurf1/2 activity, leading to degradation of Runx2. Furthermore, Smurf1-/- mice are rescued from TNF-α degradation of Runx2 [137,138]. TNF-α directly reduces alkaline phosphatase activity and reduces BMP-2/BMP-4-induced alkaline phosphatase activity when added to MC3T3 cells. Inhibiting osteoinductive signaling molecules is another mechanism in which TNF-α negatively affects osteoblasts without inhibiting Runx2 directly in a mechanism that involves NF-κB inhibition of TGF-β and Smad signaling [127,139]. BMP-2 and BMP-4, in turn, exert inhibiting effects on TNF-α by blocking TNF-α-mediated apoptosis of pluripotent mesenchymal C2C12 cells via preventing the caspase 8–mediated cleavage of Bid [140]. BMPs suppress the signal initiated by TNF-α binding to TNFR1 through stabilizing Smad signaling [140,141,142].

In line with the established role of TNF-α in inhibiting osteoblast differentiation, TNF-α also decreases Osx mRNA expression and Osx promoter activity in M3CT3 cells; however, it does not affect the brief half-life of Osx mRNA as it does with Runx2 mRNA. TNF-α inhibition of Osx promoter activity and mRNA expression is due to the activation of the MEK/ERK pathway, while p38 and JNK are not involved in this process [15]. NF-κB activation by TNF-α positively influenced Osx promoter activity in MC3T3 cells; the blockage of NF-κB did not affect TNF-α action, and overexpression of NF-κB concomitant with TNF-α treatment did not inhibit Osx transcription. This is due to the binding of NF-κB to a low-affinity enhancer element of the Osx promoter that cannot be sufficiently stimulated by the low levels of NF-κB activated by TNF-α. One study demonstrated that the regulation of NF-κB activity helps balance the effect of TNF-α in cases of severe inflammation, dampening the catabolic effects of TNF-α on bone [15]. A study in another cell model, C2C12, showed that NF-κB activation by TNF-α conferred protection against osteoblast apoptosis [140].

TNFR1 and TNFR2 activate the NF-κB pathway using TRADD and the TRAF1/TRAF2 complex. Furthermore, signaling through TRAF1/2 to activate NF-κB promotes cell survival, for the most part, while the case is not the same for TNFR1 signaling through TRADD, which promotes survival or apoptosis [133]. Which of the two receptors regulates Osx activity needs further study.

After reducing Runx2 and osterix mRNA expressions, TNF-α suppresses alkaline phosphatase and α1(I) procollagen gene expressions. The inhibition of skeletal-specific matrix proteins, osteonectin, and osteopontin is only seen with TNF-α but not with other inhibitory cytokines, such as IL-1β, which are also reported to inhibit osteogenic differentiation [114].

### 4.3. The Role of the Interaction of TNF-α and TNFRs in Osteoblast Apoptosis

TNF-α apoptotic effects on osteoblasts depend on the cell differentiation stage. While cells expressing low levels of Runx2 show inhibition of differentiation, mature osteoblasts expressing high Runx2 levels are susceptible to apoptosis by TNF-α signaling through TNFR1 [129]. TNF-α induces apoptosis in MC3T3 osteoblastic cells, an effect amplified by serum starvation [143], and induces DNA fragmentation at 20 ng/mL, as observed by BrdU-labeled DNA staining after 24 h [144]. TNF-α also reduces proliferation and induces early and late apoptosis of MSCs in a dose-dependent manner [145,146]. Other studies reported contradicting results; TNF-α had no effect on MSC viability or apoptosis, but it did decrease MC3T3 viability only after complete differentiation of osteoblasts occurred; it also increased caspase 3 activity slightly but significantly [125,130]. In another report, TNF-α treatment did not increase apoptosis or proliferation of primary human osteoblasts or the MG63 osteoblastic cell line; however, pretreatment of cells with TNF-α increased Fas expression on the cell surface, and the incubation of cells with anti-Fas IgM significantly increased Fas-mediated apoptosis of these cells [147]. Differences in osteoblast susceptibility to induction of apoptosis may be influenced by the cell differentiation stage and culture conditions in the studies, with cells in polygonal nodular osteoblast-assuming shape being more susceptible to apoptotic stimuli than less differentiated cells [122].

Transient caspase activation, in particular, caspases 2, 3, and 8 by BMP-4 is important for osteoblast differentiation, and the inhibition of these caspases leads to reduced ALP and PTH activity in MC3T3 cells. Paradoxically, triggering the caspase cascade signal is important for apoptosis; however, the critical difference in whether apoptosis or differentiation occurs is in the level of differentiation of the cells when treated with TNF-α. These results reconcile the finding that caspase activation is essential for osteoblast differentiation with the lack of apoptosis that is seen in not fully differentiated cells. Cells that have already assumed the osteoblastic phenotype are susceptible to TNF-α-induced caspase activation and apoptosis. In contrast, cells that have not yet differentiated and that are still pre-osteoblastic require the activation of the caspase pathway to assume the mature osteoblastic phenotype [144].

### 4.4. The Role of the Interaction of TNF-α and TNFRs in Osteoblast for Bone Remodeling

TNFR activation in osteoblasts generally inhibits bone formation and stimulatory bone resorption. Downstream activation of TNFRs inhibits transcription and translation of genes favoring bone formation, including α-collagen, alkaline phosphatase, osteocalcin, insulin-like growth factor 1, parathyroid hormone receptor, platelet-derived growth factor receptor, and N-cadherin genes. TNF-α induces transcriptional activation of genes in osteoblasts favoring indirect bone resorption, such as IL-6, tPA, uPA, PAI-1, gelatinases, MMPs, TIMPs, NF-κB, ICAM-1, CSF-1, GM-CSF, and G-CSF genes [130,139,148,149,150,151,152,153,154,155,156,157,158,159]. These effects on osteoblasts are elicited by activating TNFRs through TNF-α; notably, these effects were not tested on a receptor type basis, but we can only speculate through studying their downstream effectors.

The intracellular domains of TNFR1 and TNFR2 share no homology; however, TNFR1 and TNFR2 activate nearly the same pathways via different mechanisms. Upon activation, both receptors are inert and signal through TRAFs. The main difference between the two receptors is the presence of a DD only in TNFR1. The DD associates with the silencer of the death domain (SODD); upon activation of TNFR1, SODD frees the cytoplasmic domain to associate with the TRADD protein, which then recruits TRAFs to propagate the downstream signal of the receptor. The main pathways activated by TNFRs are the NF-κB, MAPK family, G protein family, and caspase pathways. Some differences are present in the selectivity and efficiency of either receptor to activate these pathways; however, their functions overlap considerably. The downstream activation of both receptors is required for the full effects seen in osteoblasts compared with effects observed upon activation of just one of the two receptors [160,161].

While TNF-α is generally inhibitory of bone formation, TNF-α shows a dual function in fracture healing. In the early stages of fracture healing, a brief but necessary inflammatory stimulus recruits osteoblast progenitors cells capable of forming osteoblasts. This observation was demonstrated in two models of fracture repair—marrow ablation and simple transverse fractures—in which TNFR-deficient mice experienced delayed healing. On day three after model establishment in wild-type mice, young osteoblasts appeared in the marrow space, while only granulation tissue appeared in the marrow cavity in TNFR-deficient mice. In the marrow ablation model in TNFR-deficient mice, type I collagen and osteocalcin mRNA expressions were reduced to 50% of levels in wild-type mice. There was a complete absence of the initial intramembranous bone formation on the periosteal surfaces in the fracture repair model. In TNFR-deficient mice, endochondral bone formation was delayed but the inhibition of osteogenesis was not observed; the intramembranous bone formation was initially completely inhibited [162].

In bone resorption induction, TNF-α is a RANKL synergist, and in bone marrow stromal cells, it induces RANKL expression through activating p38 MAPK; inhibiting p38 signaling arrests its expression. TNF-α also induces other MAPK family members, including JNK and ERK [163]. TNF-α induces IL-1 expression in osteoblasts. IL-1 acts by activating MAPKs and NF-κB, eliciting RANKL expression and promoting osteoclast formation [99,164]. TNF-α-induced RANKL and IL-1 expressions are attenuated by IL-4 via suppressing p38 activation, which inhibits osteoclast formation and bone destruction in vitro and in vivo [99, 69].

The effects of TNF-α on osteoblasts obtained from different cell sources converge towards a common outcome. Fetal calvarial cells and murine marrow stromal cells differentiation to osteoblasts is halted when cultured with TNF-α. Additionally, MC3T3 cell line treatment with TNF-α exhibits an inhibition of differentiation via decreasing Runx2 and Osx mRNA expressions and increased apoptotic changes. MSCs, in addition to experiencing inhibition of differentiation, experience reduced nodular formation and mineralization, decreased alkaline phosphatase and osteocalcin expression when signaling through TNFR1. They also experience apoptotic changes once their differentiation to the osteoblastic phenotype takes place, as MSCs apoptosis was shown to be facilitated by Runx2 expression. Cells from the C2C12 line experience apoptosis with TNF-α treatment which can be overcome with BMP-2 and BMP-4, and activation of the NF-κB pathway. Primary human osteoblasts and the MG63 osteoblastic cell line also experience apoptotic changes when treated with TNF-α due to increased Fas expression.

## 5. Osteocytes

### 5.1. TNFR1 and TNFR2 in Osteocytes

Osteocytes form from a group of osteoblasts that have become encapsulated in their secreted matrix and represent the terminally differentiated stage of the osteoblast. These cells are mechanosensing cells in the bone and perform other regulatory functions, including bone remodeling and maintaining mineral homeostasis [164]. Osteocytes respond and express inflammatory cytokines in a positive feedback loop, which creates an environment through which inflammatory diseases can quickly propagate, giving osteocytes an essential role as regulators of inflammation [165]. Although osteocytes form more than 95% of bone cells, the understanding of the interactions of their surface receptors with signaling molecules and cytokines lags behind that of osteoclasts and osteoblasts because osteocytes are embedded in a hard-to-access mineralized matrix and research has thus been limited. Cell lines and transgenic mouse models provide an alternative to primary cells for the study of osteocytes; nevertheless, the exact dynamics of how TNF-α signals through its two receptors in osteocytes have not been reported. Therefore, we report on the effect of TNF-α on osteocytes.

Osteocytes express both TNFRs, and the effects of TNF-α on osteocytes are similar to those on osteoblasts. TNF-α promotes osteocyte apoptosis and steers osteocyte control over bone remodeling in a negative net balance. In addition, and per osteocyte involvement in propagating inflammation, TNF-α is involved in osteocytic expression and the withholding of signals that exacerbate inflammation [20,165].

### 5.2. The Role of the Interaction of TNF-α and TNFRs in Osteocyte Apoptosis

Osteocyte apoptosis is a function of two distinct processes. The first preserves the integrity and strength of bone by removing senile osteocytes, which promotes self-renewal of bone tissue [166,167]. The second is a reaction to an array of pathological conditions, including unloading [168,169], microdamage and fatigue [170], glucocorticoid excess [171], estrogen deficiency [172], inflammatory bowel disease [173,174], and infections [175]. In all of these conditions, a correlation between increased TNF-α levels and osteocyte apoptosis is observed.

TNF-α increased apoptosis in the MLO-Y4 osteocytic cell line from baseline to 13%–18% (depending on the apoptotic test used), which can be attenuated using the CD40 ligand [176,177]. Another study suggested that TNF-α treatment did not induce apoptosis, as it did not significantly increase the activities of caspase3/7, two major caspases required for apoptosis [178].

### 5.3. The Role of the Interaction of TNF-α and TNFRs in Osteocytes for Bone Remodeling

Osteocytes express both TNFR1 and TNFR2. A wealth of investigators has established that TNF-α is mostly correlated with osteocytic-induced bone loss, either directly via RANKL expression in diseases of inflammatory bone loss, such as rheumatoid arthritis and periodontitis [23,179,180], or indirectly, via inducing osteocyte apoptosis, which releases osteoclast formation promoting factors RANKL, IL-6 and endothelial intercellular adhesion molecule-1 [181,182,183].

TNF-α works directly on osteocytes to increase their osteoclastogenic ability via RANKL expression; osteocytes treated with TNF-α induce the formation of osteoclasts in vitro from TNFR-deficient osteoclast precursors. In mice lacking TNFRs, osteocyte RANKL expression, osteoclast formation, and the OTM distance are significantly decreased compared with observations in wild-type mice. TNF-α signaling in the osteocyte activates the NF-κB pathway and signals RANKL mRNA expression through ERK1/2 and p38 MAPKs [23].

TNF-α also inhibited pulsatile fluid flow-stimulated NO production and decreased pulsatile fluid flow-stimulated (Ca^2+^) intracellular levels by MLO-Y4, and reduced the F-actin content to 63% of the control values [178]. This is important because osteocyte shape depends on the actin cytoskeletal organization [183], and osteocyte shape has been reported to be different among different bone pathologies, probably due to the differential response to external loading [181]. Whether this has therapeutic implications is not yet clear.

Interestingly, TNF-α can induce osteocytic bone resorption independent of RANKL. Kogianni et al. demonstrated that osteocyte apoptotic antibodies induced bone resorption in vitro and in vivo. The co-administration of the TNF-α neutralizing antibody with osteocyte apoptotic antibodies reduced bone resorption, an effect not seen with OPG or when osteocytes are treated with TNF-α neutralizing antibody [184]. TNF-α also promotes the expression of sclerostin, an antagonist of Wnt/β-catenin signaling that regulates osteoblast differentiation, which is blocked by blocking NF-κB activation [185]. Furthermore, the antagonist infliximab was shown to reduce RANKL and sclerostin expression in osteocytes in diabetic rats with periodontitis [180]. Sclerostin expression in conjunction with RANKL expression by osteocytes results in a net negative balance during bone remodeling and ends with decreased bone mass [86,185,186]. TNF-α also contributes to high sclerostin expression in MLO-Y4 cells induced by high glucose treatment, as TNF-α siRNA reduces SOST mRNA expression, implicating TNF signaling in and by osteocytes to dysglycemia [187]. TNF-α increases FGF23 mRNA expression in the IDG-SW3 osteocytic cell line via NF-κB and is independent of MAPKs and reduces Phex, Dmp1, and Enpp1 mRNA expressions. This implicates TNF-α signaling in FGF expression by osteocytes with the regulation of phosphate and vitamin D metabolism, as well as bone mineralization [188,189]. Osteocytes are involved in the pathogenesis of several inflammatory systemic conditions, including inflammatory bowel disease [173], spinal cord injury [190], rheumatoid arthritis [179], and psoriasis [191]. However, a direct cause-effect relationship between TNF-α and these conditions has not yet been established.

Osteocytes seem to be either involved in propagating inflammation or are heavily affected by inflammation. TNF-α signaling in osteocytes relative to its functions in other bone cells is not completely understood and needs further clarification. TNF-α is a ubiquitous inflammatory cytokine, and its effect on osteocytes is worth further study. TNF-α signaling is also vital for initiating fracture healing, and osteocytes supply cancellous bone with the RANKL needed for bone remodeling in the mature skeleton. How TNF-α signaling in the osteocyte affects local and systemic inflammatory conditions should be examined in further research. Whether osteocytes have potential therapeutic value needs more attention.

**Figure 1:**
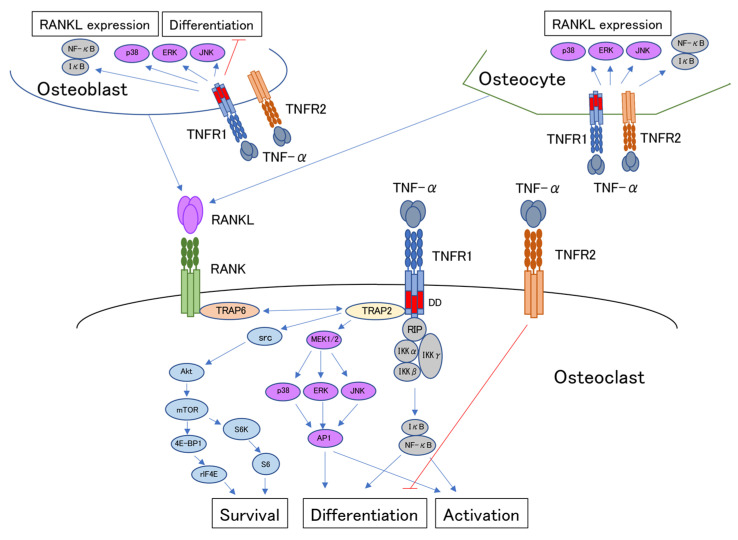
Schematic of the interaction of tumor necrosis factor-α (TNF-α) and tumor necrosis factor receptors 1 and 2 (TNFR1 and TNFR2) in bone-related cells, osteoclasts, osteoblasts, and osteocytes. TNF-α induces osteoclast formation and activates transcription factor AP-1 through phosphorylation of MAPKs (ERK, p38, and JNK) and induces activation of transcription factor NF-κB through phosphorylation of IκB in osteoclast precursors via TNFR1. On the other hand, TNF-α inhibits osteoclast differentiation via TNFR2. TNF-α induced an anti-apoptotic effect by mTOR signaling by activated Src and Akt. mTOR regulates protein translation through S6K, 4E-BP1, S6, and elF4E, and inhibits osteoclast apoptosis. TNF-α induces RANKL expression by MAPKs and NF-κB activation in osteoblasts but inhibits osteoclast differentiation via TNFR1. TNF-α induces RANKL expression by activation of MAPKs and NF-κB in osteocytes.

TNFR1 and TNFR2 signaling pathways activated by TNF-α have been investigated in osteoclasts, osteoblasts, and osteocytes. Finally, we summarize how these cells interact to progress bone resorption, as shown in Figure 1.

## 6. Conclusions

The well-documented role of TNF-α in bone inflammatory diseases is to promote bone resorption and contribute to disease progression. TNF-α directly induces osteoclast formation via TNFR1 but not TNFR2 and indirectly induces osteoclast formation by TNF-α-induced RANKL expression in osteoblasts and osteocytes. In this way, TNF-α induces bone resorption events from multiple directions. TNFR1 and TNFR2 signaling pathways activated by TNF-α have been investigated in osteoclasts, osteoblasts, and osteocytes, with both receptors capable of activating MAPKs phosphorylation and activating the NF-κB pathway through different adaptor proteins. TNFRs signaling may induce apoptotic or survival signals depending on the type of cell involved, and the receptor activated, while signaling in osteoclasts is generally proliferative, and signaling in osteoblasts and osteocytes is inhibitory. Few studies have examined the effects of the TNF-α and TNFRs interaction in bone-related cells. Further studies are required to elucidate the relationship between TNF-α and TNFRs.

## Abbreviations

TNF-α	tumor necrosis factor-α
TNFR1	TNF receptor-1
TNFR2	TNF receptor-2
RANKL	receptor activator of nuclear factor κB ligand
LT-α	lymphotoxin alpha
TNFRSF	tumor necrosis factor receptor superfamily
TNFSF	tumor necrosis factor superfamily
CRD	cysteine-rich domain
TRAF	TNF receptor-associated factors
DD	death domain
RIPK1	receptor-interacting protein kinase 1
TRADD	TNFR1-associated death domain
FADD	Fas-associated death domain
M-CSF	macrophage colony-stimulating factor
OTM	orthodontic tooth movement
MAPK	mitogen-activated protein kinase
NFATc1	nuclear factor of activated T-cell c1
OSCAR	osteoclast-associated receptor
ITAM	immunoreceptor tyrosine-based activation motifs
mTOR	mammalian target of rapamycin
LPL	L-plastin
MSCs	mesenchymal stem cells
Osx	osterix
SODD	silencer of the death domain

## References

[1] Hadjidakis D.J., Androulakis I.I. (2006). Bone remodeling. Ann. N. Y. Acad. Sci..

[2] Kim J.M., Lin C., Stavre Z., Greenblatt M.B., Shim J.H. (2020). Osteoblast-Osteoclast Communication and Bone Homeostasis. Cells.

[3] Tresguerres F.G.F., Torres J., Lopez-Quiles J., Hernandez G., Vega J.A., Tresguerres I.F. (2020). The osteocyte: A multifunctional cell within the bone. Ann. Anat..

[4] Uda Y., Azab E., Sun N., Shi C., Pajevic P.D. (2017). Osteocyte Mechanobiology. Curr. Osteoporos. Rep..

[5] Tracey K.J., Cerami A. (1993). Tumor necrosis factor, other cytokines and disease. Annu. Rev. Cell Biol..

[6] Teitelbaum S.L. (2000). Bone resorption by osteoclasts. Science.

[7] Hienz S.A., Paliwal S., Ivanovski S. (2015). Mechanisms of Bone Resorption in Periodontitis. J. Immunol. Res..

[8] Goodman S.B., Gallo J. (2019). Periprosthetic Osteolysis: Mechanisms, Prevention and Treatment. J. Clin. Med..

[9] Aureal M., Machuca-Gayet I., Coury F. (2020). Rheumatoid Arthritis in the View of Osteoimmunology. Biomolecules.

[10] Wong M., Ziring D., Korin Y., Desai S., Kim S., Lin J., Gjertson D., Braun J., Reed E., Singh R.R. (2008). TNFalpha blockade in human diseases: Mechanisms and future directions. Clin. Immunol..

[11] Sakthiswary R., Das S. (2013). The effects of TNF alpha antagonist therapy on bone metabolism in rheumatoid arthritis: A systematic review. Curr. Drug Targets.

[12] Huang W., Yang S., Shao J., Li Y.P. (2007). Signaling and transcriptional regulation in osteoblast commitment and differentiation. Front. Biosci..

[13] Lee W.C., Guntur A.R., Long F., Rosen C.J. (2017). Energy Metabolism of the Osteoblast: Implications for Osteoporosis. Endocr. Rev..

[14] Lane N.E. (2019). Glucocorticoid-Induced Osteoporosis: New Insights into the Pathophysiology and Treatments. Curr. Osteoporos..

[15] Lu X., Gilbert L., He X., Rubin J., Nanes M.S. (2006). Transcriptional regulation of the osterix (Osx, Sp7) promoter by tumor necrosis factor identifies disparate effects of mitogen-activated protein kinase and NF kappa B pathways. Biol. Chem..

[16] Franz-Odendaal T.A., Hall B.K., Witten P.E. (2006). Buried alive: How osteoblasts become osteocytes. Dev. Dyn..

[17] Holmbeck K., Bianco P., Pidoux I., Inoue S., Billinghurst R.C., Wu W., Chrysovergis K., Yamada S., Birkedal-Hansen H., Poole A.R. (2005). The metalloproteinase MT1-MMP is required for normal development and maintenance of osteocyte processes in bone. J. Cell Sci..

[18] Bonewald L.F. (2007). Osteocytes as dynamic multifunctional cells. Ann. N. Y. Acad. Sci..

[19] Bonewald L.F. (2011). The amazing osteocyte. J. Bone Miner. Res..

[20] Kitaura H., Marahleh A., Ohori F., Noguchi T., Shen W.R., Qi J., Nara Y., Pramusita A., Kinjo R., Mizoguchi I. (2020). Osteocyte-Related Cytokines Regulate Osteoclast Formation and Bone Resorption. Int. J. Mol. Sci..

[21] Nakashima T., Hayashi M., Fukunaga T., Kurata K., Oh-Hora M., Feng J.Q., Bonewald L.F., Kodama T., Wutz A., Wagner E.F. (2011). Evidence for osteocyte regulation of bone homeostasis through RANKL expression. Nat. Med..

[22] Xiong J., Piemontese M., Onal M., Campbell J., Goellner J.J., Dusevich V., Bonewald L., Manolagas S.C., O’Brien C.A. (2015). Osteocytes, not Osteoblasts or Lining Cells, are the Main Source of the RANKL Required for Osteoclast Formation in Remodeling Bone. PLoS ONE.

[23] Marahleh A., Kitaura H., Ohori F., Kishikawa A., Ogawa S., Shen W.R., Qi J., Noguchi T., Nara Y., Mizoguchi I. (2019). TNF-alpha Directly Enhances Osteocyte RANKL Expression and Promotes Osteoclast Formation. Front. Immunol..

[24] Loetscher H., Schlaeger E.J., Lahm H.W., Pan Y.C., Lesslauer W., Brockhaus M. (1990). Purification and partial amino acid sequence analysis of two distinct tumor necrosis factor receptors from HL60 cells. J. Biol. Chem..

[25] Dostert C., Grusdat M., Letellier E., Brenner D. (2019). The TNF Family of Ligands and Receptors: Communication Modules in the Immune System and Beyond. Physiol. Rev..

[26] Dembic Z., Loetscher H., Gubler U., Pan Y.C., Lahm H.W., Gentz R., Brockhaus M., Lesslauer W. (1990). Two human TNF receptors have similar extracellular, but distinct intracellular, domain sequences. Cytokine.

[27] Jacobsen F.W., Rothe M., Rusten L., Goeddel D.V., Smeland E.B., Veiby O.P., Slordal L., Jacobsen S.E. (1994). Role of the 75-kDa tumor necrosis factor receptor: Inhibition of early hematopoiesis. Proc. Natl. Acad. Sci. USA.

[28] Bodmer J.L., Schneider P., Tschopp J. (2002). The molecular architecture of the TNF superfamily. Trends Biochem. Sci..

[29] Su Z., Wu Y. (2020). A Systematic Test of Receptor Binding Kinetics for Ligands in Tumor Necrosis Factor Superfamily by Computational Simulations. Int. J. Mol. Sci..

[30] Tartaglia L.A., Goeddel D.V. (1992). Two TNF receptors. Immunol. Today.

[31] Park Y.H., Jeong M.S., Jang S.B. (2016). Structural insights of homotypic interaction domains in the ligand-receptor signal transduction of tumor necrosis factor (TNF). BMB Rep..

[32] Al-Lamki R.S., Mayadas T.N. (2015). TNF receptors: Signaling pathways and contribution to renal dysfunction. Kidney Int..

[33] Rothe M., Sarma V., Dixit V.M., Goeddel D.V. (1995). TRAF2-mediated activation of NF-kappa B by TNF receptor 2 and CD40. Science.

[34] Rothe M., Wong S.C., Henzel W.J., Goeddel D.V. (1994). A novel family of putative signal transducers associated with the cytoplasmic domain of the 75 kDa tumor necrosis factor receptor. Cell.

[35] Tartaglia L.A., Ayres T.M., Wong G.H., Goeddel D.V. (1993). A novel domain within the 55 kd TNF receptor signals cell death. Cell.

[36] Idress M., Milne B.F., Thompson G.S., Trembleau L., Jaspars M., Houssen W.E. (2020). Structure-Based Design, Synthesis and Bioactivity of a New Anti-TNFalpha Cyclopeptide. Molecules.

[37] Matsumoto M., Fu Y.X., Molina H., Chaplin D.D. (1997). Lymphotoxin-alpha-deficient and TNF receptor-I-deficient mice define developmental and functional characteristics of germinal centers. Immunol. Rev..

[38] Jacobs M., Brown N., Allie N., Chetty K., Ryffel B. (2000). Tumor necrosis factor receptor 2 plays a minor role for mycobacterial immunity. Pathobiology.

[39] Endres R., Luz A., Schulze H., Neubauer H., Futterer A., Holland S.M., Wagner H., Pfeffer K. (1997). Listeriosis in p47(phox-/-) and TRp55-/- mice: Protection despite absence of ROI and susceptibility despite presence of RNI. Immunity.

[40] Peschon J.J., Torrance D.S., Stocking K.L., Glaccum M.B., Otten C., Willis C.R., Charrier K., Morrissey P.J., Ware C.B., Mohler K.M. (1998). TNF receptor-deficient mice reveal divergent roles for p55 and p75 in several models of inflammation. J. Immunol..

[41] Morris D.R., Ansar M., Ivanciuc T., Qu Y., Casola A., Garofalo R.P. (2020). Selective Blockade of TNFR1 Improves Clinical Disease and Bronchoconstriction in Experimental RSV Infection. Viruses.

[42] Beldi G., Khosravi M., Abdelgawad M.E., Salomon B.L., Uzan G., Haouas H., Naserian S. (2020). TNFalpha/TNFR2 signaling pathway: An active immune checkpoint for mesenchymal stem cell immunoregulatory function. Stem Cell Res. Ther..

[43] Yang S., Wang J., Brand D.D., Zheng S.G. (2018). Role of TNF-TNF Receptor 2 Signal in Regulatory T Cells and Its Therapeutic Implications. Front. Immunol..

[44] Shamdani S., Uzan G., Naserian S. (2020). TNFalpha-TNFR2 signaling pathway in control of the neural stem/progenitor cell immunosuppressive effect: Different experimental approaches to assess this hypothetical mechanism behind their immunological function. Stem Cell Res. Ther..

[45] Faustman D.L., Davis M. (2013). TNF Receptor 2 and Disease: Autoimmunity and Regenerative Medicine. Front. Immunol..

[46] Leclerc M., Naserian S., Pilon C., Thiolat A., Martin G.H., Pouchy C., Dominique C., Belkacemi Y., Charlotte F., Maury S. (2016). Control of GVHD by regulatory T cells depends on TNF produced by T cells and TNFR2 expressed by regulatory T cells. Blood.

[47] Ticha O., Moos L., Wajant H., Bekeredjian-Ding I. (2017). Expression of Tumor Necrosis Factor Receptor 2 Characterizes TLR9-Driven Formation of Interleukin-10-Producing B Cells. Front. Immunol..

[48] Chen Z., Palmer T.D. (2013). Differential roles of TNFR1 and TNFR2 signaling in adult hippocampal neurogenesis. Brain Behav. Immun..

[49] Sheng Y., Li F., Qin Z. (2018). TNF Receptor 2 Makes Tumor Necrosis Factor a Friend of Tumors. Front. Immunol..

[50] Tartaglia L.A., Weber R.F., Figari I.S., Reynolds C., Palladino M.A., Goeddel D.V. (1991). The two different receptors for tumor necrosis factor mediate distinct cellular responses. Proc. Natl. Acad. Sci. USA.

[51] Chan F.K., Lenardo M.J. (2000). A crucial role for p80 TNF-R2 in amplifying p60 TNF-R1 apoptosis signals in T lymphocytes. Eur. J. Immunol..

[52] Nishitoh H., Saitoh M., Mochida Y., Takeda K., Nakano H., Rothe M., Miyazono K., Ichijo H. (1998). ASK1 is essential for JNK/SAPK activation by TRAF2. Mol. Cell.

[53] Segueni N., Benmerzoug S., Rose S., Gauthier A., Bourigault M.L., Reverchon F., Philippeau A., Erard F., Le Bert M., Bouscayrol H. (2016). Innate myeloid cell TNFR1 mediates first line defence against primary Mycobacterium tuberculosis infection. Sci. Rep..

[54] Wroblewski R., Armaka M., Kondylis V., Pasparakis M., Walczak H., Mittrucker H.W., Schramm C., Lohse A.W., Kollias G., Ehlken H. (2016). Opposing role of tumor necrosis factor receptor 1 signaling in T cell-mediated hepatitis and bacterial infection in mice. Hepatology.

[55] Chen Q., Hou T., Wu X., Luo F., Xie Z., Xu J. (2016). Knockdown of TNFR1 Suppresses Expression of TLR2 in the Cellular Response to Staphylococcus aureus Infection. Inflammation.

[56] Ruby J., Bluethmann H., Peschon J.J. (1997). Antiviral activity of tumor necrosis factor (TNF) is mediated via p55 and p75 TNF receptors. J. Exp. Med..

[57] Ebach D.R., Riehl T.E., Stenson W.F. (2005). Opposing effects of tumor necrosis factor receptor 1 and 2 in sepsis due to cecal ligation and puncture. Shock.

[58] Yasuda H., Shima N., Nakagawa N., Yamaguchi K., Kinosaki M., Mochizuki S., Tomoyasu A., Yano K., Goto M., Murakami A. (1998). Osteoclast differentiation factor is a ligand for osteoprotegerin/osteoclastogenesis-inhibitory factor and is identical to TRANCE/RANKL. Proc. Natl. Acad. Sci. USA.

[59] Lacey D.L., Timms E., Tan H.L., Kelley M.J., Dunstan C.R., Burgess T., Elliott R., Colombero A., Elliott G., Scully S. (1998). Osteoprotegerin ligand is a cytokine that regulates osteoclast differentiation and activation. Cell.

[60] Ross F.P., Teitelbaum S.L. (2005). alphavbeta3 and macrophage colony-stimulating factor: Partners in osteoclast biology. Immunol. Rev..

[61] Kobayashi K., Takahashi N., Jimi E., Udagawa N., Takami M., Kotake S., Nakagawa N., Kinosaki M., Yamaguchi K., Shima N. (2000). Tumor necrosis factor alpha stimulates osteoclast differentiation by a mechanism independent of the ODF/RANKL-RANK interaction. J. Exp. Med..

[62] Azuma Y., Kaji K., Katogi R., Takeshita S., Kudo A. (2000). Tumor necrosis factor-alpha induces differentiation of and bone resorption by osteoclasts. J. Biol. Chem..

[63] Lam J., Takeshita S., Barker J.E., Kanagawa O., Ross F.P., Teitelbaum S.L. (2000). TNF-alpha induces osteoclastogenesis by direct stimulation of macrophages exposed to permissive levels of RANK ligand. J. Clin. Investig..

[64] Li P., Schwarz E.M., O’Keefe R.J., Ma L., Boyce B.F., Xing L. (2004). RANK signaling is not required for TNFalpha-mediated increase in CD11(hi) osteoclast precursors but is essential for mature osteoclast formation in TNFalpha-mediated inflammatory arthritis. J. Bone Miner. Res..

[65] Kim N., Kadono Y., Takami M., Lee J., Lee S.H., Okada F., Kim J.H., Kobayashi T., Odgren P.R., Nakano H. (2005). Osteoclast differentiation independent of the TRANCE-RANK-TRAF6 axis. J. Exp. Med..

[66] Udagawa N., Takahashi N., Akatsu T., Tanaka H., Sasaki T., Nishihara T., Koga T., Martin T.J., Suda T. (1990). Origin of osteoclasts: Mature monocytes and macrophages are capable of differentiating into osteoclasts under a suitable microenvironment prepared by bone marrow-derived stromal cells. Proc. Natl. Acad. Sci. USA.

[67] Kong Y.Y., Feige U., Sarosi I., Bolon B., Tafuri A., Morony S., Capparelli C., Li J., Elliott R., McCabe S. (1999). Activated T cells regulate bone loss and joint destruction in adjuvant arthritis through osteoprotegerin ligand. Nature.

[68] Kitaura H., Zhou P., Kim H.J., Novack D.V., Ross F.P., Teitelbaum S.L. (2005). M-CSF mediates TNF-induced inflammatory osteolysis. J. Clin. Investig..

[69] Fujii T., Kitaura H., Kimura K., Hakami Z.W., Takano-Yamamoto T. (2012). IL-4 inhibits TNF-alpha-mediated osteoclast formation by inhibition of RANKL expression in TNF-alpha-activated stromal cells and direct inhibition of TNF-alpha-activated osteoclast precursors via a T-cell-independent mechanism in vivo. Bone.

[70] Kohara H., Kitaura H., Fujimura Y., Yoshimatsu M., Morita Y., Eguchi T., Masuyama R., Yoshida N. (2011). IFN-gamma directly inhibits TNF-alpha-induced osteoclastogenesis in vitro and in vivo and induces apoptosis mediated by Fas/Fas ligand interactions. Immunol. Lett..

[71] Morita Y., Kitaura H., Yoshimatsu M., Fujimura Y., Kohara H., Eguchi T., Yoshida N. (2010). IL-18 inhibits TNF-alpha-induced osteoclastogenesis possibly via a T cell-independent mechanism in synergy with IL-12 in vivo. Calcif. Tissue Int..

[72] Ohori F., Kitaura H., Ogawa S., Shen W.R., Qi J., Noguchi T., Marahleh A., Nara Y., Pramusita A., Mizoguchi I. (2020). IL-33 Inhibits TNF-alpha-Induced Osteoclastogenesis and Bone Resorption. Int. J. Mol. Sci..

[73] Yoshimatsu M., Kitaura H., Fujimura Y., Eguchi T., Kohara H., Morita Y., Yoshida N. (2009). IL-12 inhibits TNF-alpha induced osteoclastogenesis via a T cell-independent mechanism in vivo. Bone.

[74] Kitaura H., Sands M.S., Aya K., Zhou P., Hirayama T., Uthgenannt B., Wei S., Takeshita S., Novack D.V., Silva M.J. (2004). Marrow stromal cells and osteoclast precursors differentially contribute to TNF-alpha-induced osteoclastogenesis in vivo. J. Immunol..

[75] Andrade I., Silva T.A., Silva G.A., Teixeira A.L., Teixeira M.M. (2007). The role of tumor necrosis factor receptor type 1 in orthodontic tooth movement. J. Dent. Res..

[76] Basaran G., Ozer T., Kaya F.A., Kaplan A., Hamamci O. (2006). Interleukine-1beta and tumor necrosis factor-alpha levels in the human gingival sulcus during orthodontic treatment. Angle Orthod..

[77] Garlet T.P., Coelho U., Silva J.S., Garlet G.P. (2007). Cytokine expression pattern in compression and tension sides of the periodontal ligament during orthodontic tooth movement in humans. Eur. J. Oral Sci..

[78] Benjakul S., Unat B., Thammanichanon P., Leethanakul C. (2020). Vibration synergistically enhances IL-1beta and TNF-alpha in compressed human periodontal ligament cells in the frequency-dependent manner. J. Oral Biol. Craniofac. Res..

[79] Jayaprakash P.K., Basavanna J.M., Grewal H., Modi P., Sapawat P., Bohara P.D. (2019). Elevated levels of Interleukin (IL)-1beta, IL-6, tumor necrosis factor-alpha, epidermal growth factor, and beta2-microglobulin levels in gingival crevicular fluid during human Orthodontic tooth movement (OTM). J. Family Med. Prim. Care.

[80] Ren Y., Hazemeijer H., de Haan B., Qu N., de Vos P. (2007). Cytokine profiles in crevicular fluid during orthodontic tooth movement of short and long durations. J. Periodontol..

[81] Yoshimatsu M., Shibata Y., Kitaura H., Chang X., Moriishi T., Hashimoto F., Yoshida N., Yamaguchi A. (2006). Experimental model of tooth movement by orthodontic force in mice and its application to tumor necrosis factor receptor-deficient mice. J. Bone Miner. Metab..

[82] Kitaura H., Yoshimatsu M., Fujimura Y., Eguchi T., Kohara H., Yamaguchi A., Yoshida N. (2008). An anti-c-Fms antibody inhibits orthodontic tooth movement. J. Dent. Res..

[83] Ogawa S., Kitaura H., Kishikawa A., Qi J., Shen W.R., Ohori F., Noguchi T., Marahleh A., Nara Y., Ochi Y. (2019). TNF-alpha is responsible for the contribution of stromal cells to osteoclast and odontoclast formation during orthodontic tooth movement. PLoS ONE.

[84] Marahleh A., Kitaura H., Ohori F., Noguchi T., Nara Y., Pramusita A., Kinjo R., Ma J., Kanou K., Mizoguchi I. (2021). Effect of TNF-alpha on osteocyte RANKL expression during orthodontic tooth movement. J. Dent. Sci..

[85] Noguchi T., Kitaura H., Ogawa S., Qi J., Shen W.R., Ohori F., Marahleh A., Nara Y., Pramusita A., Mizoguchi I. (2020). TNF-alpha stimulates the expression of RANK during orthodontic tooth movement. Arch. Oral Biol..

[86] Ohori F., Kitaura H., Marahleh A., Kishikawa A., Ogawa S., Qi J., Shen W.R., Noguchi T., Nara Y., Mizoguchi I. (2019). Effect of TNF-alpha-Induced Sclerostin on Osteocytes during Orthodontic Tooth Movement. J. Immunol. Res..

[87] Abu-Amer Y., Erdmann J., Alexopoulou L., Kollias G., Ross F.P., Teitelbaum S.L. (2000). Tumor necrosis factor receptors types 1 and 2 differentially regulate osteoclastogenesis. J. Biol. Chem..

[88] Takayanagi H., Kim S., Taniguchi T. (2002). Signaling crosstalk between RANKL and interferons in osteoclast differentiation. Arthritis Res..

[89] Teitelbaum S.L., Ross F.P. (2003). Genetic regulation of osteoclast development and function. Nat. Rev. Genet..

[90] Iotsova V., Caamano J., Loy J., Yang Y., Lewin A., Bravo R. (1997). Osteopetrosis in mice lacking NF-kappaB1 and NF-kappaB2. Nat. Med..

[91] Grigoriadis A.E., Wang Z.Q., Cecchini M.G., Hofstetter W., Felix R., Fleisch H.A., Wagner E.F. (1994). c-Fos: A key regulator of osteoclast-macrophage lineage determination and bone remodeling. Science.

[92] Naito A., Azuma S., Tanaka S., Miyazaki T., Takaki S., Takatsu K., Nakao K., Nakamura K., Katsuki M., Yamamoto T. (1999). Severe osteopetrosis, defective interleukin-1 signalling and lymph node organogenesis in TRAF6-deficient mice. Genes Cells.

[93] Lomaga M.A., Yeh W.C., Sarosi I., Duncan G.S., Furlonger C., Ho A., Morony S., Capparelli C., Van G., Kaufman S. (1999). TRAF6 deficiency results in osteopetrosis and defective interleukin-1, CD40, and LPS signaling. Genes Dev..

[94] Takayanagi H., Kim S., Koga T., Nishina H., Isshiki M., Yoshida H., Saiura A., Isobe M., Yokochi T., Inoue J. (2002). Induction and activation of the transcription factor NFATc1 (NFAT2) integrate RANKL signaling in terminal differentiation of osteoclasts. Dev. Cell.

[95] Negishi-Koga T., Takayanagi H. (2009). Ca^2+^-NFATc1 signaling is an essential axis of osteoclast differentiation. Immunol. Rev..

[96] Koga T., Inui M., Inoue K., Kim S., Suematsu A., Kobayashi E., Iwata T., Ohnishi H., Matozaki T., Kodama T. (2004). Costimulatory signals mediated by the ITAM motif cooperate with RANKL for bone homeostasis. Nature.

[97] Kaji K., Katogi R., Azuma Y., Naito A., Inoue J.I., Kudo A. (2001). Tumor necrosis factor alpha-induced osteoclastogenesis requires tumor necrosis factor receptor-associated factor 6. J. Bone Miner. Res..

[98] Kanazawa K., Kudo A. (2005). TRAF2 is essential for TNF-alpha-induced osteoclastogenesis. J. Bone Miner. Res..

[99] Wei S., Kitaura H., Zhou P., Ross F.P., Teitelbaum S.L. (2005). IL-1 mediates TNF-induced osteoclastogenesis. J. Clin. Investig..

[100] Bai S., Kitaura H., Zhao H., Chen J., Muller J.M., Schule R., Darnay B., Novack D.V., Ross F.P., Teitelbaum S.L. (2005). FHL2 inhibits the activated osteoclast in a TRAF6-dependent manner. J. Clin. Investig..

[101] Lee S.E., Chung W.J., Kwak H.B., Chung C.H., Kwack K.B., Lee Z.H., Kim H.H. (2001). Tumor necrosis factor-alpha supports the survival of osteoclasts through the activation of Akt and ERK. J. Biol. Chem..

[102] Barkett M., Gilmore T.D. (1999). Control of apoptosis by Rel/NF-kappaB transcription factors. Oncogene.

[103] Glantschnig H., Fisher J.E., Wesolowski G., Rodan G.A., Reszka A.A. (2003). M-CSF, TNFalpha and RANK ligand promote osteoclast survival by signaling through mTOR/S6 kinase. Cell Death Differ..

[104] Fuller K., Murphy C., Kirstein B., Fox S.W., Chambers T.J. (2002). TNFalpha potently activates osteoclasts, through a direct action independent of and strongly synergistic with RANKL. Endocrinology.

[105] Chellaiah M.A. (2005). Regulation of actin ring formation by rho GTPases in osteoclasts. J. Biol. Chem..

[106] Lee E.J., Kim J.L., Gong J.H., Park S.H., Kang Y.H. (2015). Inhibition of osteoclast activation by phloretin through disturbing alphavbeta3 integrin-c-Src pathway. BioMed Res. Int..

[107] Schmidt S., Nakchbandi I., Ruppert R., Kawelke N., Hess M.W., Pfaller K., Jurdic P., Fassler R., Moser M. (2011). Kindlin-3-mediated signaling from multiple integrin classes is required for osteoclast-mediated bone resorption. J. Cell Biol..

[108] Ma T., Sadashivaiah K., Madayiputhiya N., Chellaiah M.A. (2010). Regulation of sealing ring formation by L-plastin and cortactin in osteoclasts. J. Biol. Chem..

[109] Schaffner-Reckinger E., Machado R.A.C. (2020). The actin-bundling protein L-plastin-A double-edged sword: Beneficial for the immune response, maleficent in cancer. Int. Rev. Cell Mol. Biol..

[110] Lommel M.J., Trairatphisan P., Gabler K., Laurini C., Muller A., Kaoma T., Vallar L., Sauter T., Schaffner-Reckinger E. (2016). L-plastin Ser5 phosphorylation in breast cancer cells and in vitro is mediated by RSK downstream of the ERK/MAPK pathway. FASEB J..

[111] Chellaiah M.A. (2021). L-Plastin Phosphorylation: Possible Regulation by a TNFR1 Signaling Cascade in Osteoclasts. Cells.

[112] Tavassoli M., Crosby W.H. (1968). Transplantation of marrow to extramedullary sites. Science.

[113] Bianco P., Robey P.G., Simmons P.J. (2008). Mesenchymal stem cells: Revisiting history, concepts, and assays. Cell Stem Cell.

[114] Lacey D.C., Simmons P.J., Graves S.E., Hamilton J.A. (2009). Proinflammatory cytokines inhibit osteogenic differentiation from stem cells: Implications for bone repair during inflammation. Osteoarthr. Cartil..

[115] Pittenger M.F., Mackay A.M., Beck S.C., Jaiswal R.K., Douglas R., Mosca J.D., Moorman M.A., Simonetti D.W., Craig S., Marshak D.R. (1999). Multilineage potential of adult human mesenchymal stem cells. Science.

[116] Baddoo M., Hill K., Wilkinson R., Gaupp D., Hughes C., Kopen G.C., Phinney D.G. (2003). Characterization of mesenchymal stem cells isolated from murine bone marrow by negative selection. J. Cell Biochem..

[117] Ducy P., Zhang R., Geoffroy V., Ridall A.L., Karsenty G. (1997). Osf2/Cbfa1: A transcriptional activator of osteoblast differentiation. Cell.

[118] Komori T., Yagi H., Nomura S., Yamaguchi A., Sasaki K., Deguchi K., Shimizu Y., Bronson R.T., Gao Y.H., Inada M. (1997). Targeted disruption of Cbfa1 results in a complete lack of bone formation owing to maturational arrest of osteoblasts. Cell.

[119] Otto F., Thornell A.P., Crompton T., Denzel A., Gilmour K.C., Rosewell I.R., Stamp G.W., Beddington R.S., Mundlos S., Olsen B.R. (1997). Cbfa1, a candidate gene for cleidocranial dysplasia syndrome, is essential for osteoblast differentiation and bone development. Cell.

[120] Nakashima K., de Crombrugghe B. (2003). Transcriptional mechanisms in osteoblast differentiation and bone formation. Trends Genet..

[121] Nakashima K., Zhou X., Kunkel G., Zhang Z., Deng J.M., Behringer R.R., de Crombrugghe B. (2002). The novel zinc finger-containing transcription factor osterix is required for osteoblast differentiation and bone formation. Cell.

[122] Bu R., Borysenko C.W., Li Y., Cao L., Sabokbar A., Blair H.C. (2003). Expression and function of TNF-family proteins and receptors in human osteoblasts. Bone.

[123] Faustman D., Davis M. (2010). TNF receptor 2 pathway: Drug target for autoimmune diseases. Nat. Rev. Drug Discov..

[124] Bocker W., Docheva D., Prall W.C., Egea V., Pappou E., Rossmann O., Popov C., Mutschler W., Ries C., Schieker M. (2008). IKK-2 is required for TNF-alpha-induced invasion and proliferation of human mesenchymal stem cells. J. Mol. Med..

[125] Gilbert L.C., Rubin J., Nanes M.S. (2005). The p55 TNF receptor mediates TNF inhibition of osteoblast differentiation independently of apoptosis. Am. J. Physiol. Endocrinol. Metab..

[126] MacEwan D.J. (2002). TNF receptor subtype signalling: Differences and cellular consequences. Cell Signal..

[127] Li Y., Li A., Strait K., Zhang H., Nanes M.S., Weitzmann M.N. (2007). Endogenous TNFalpha lowers maximum peak bone mass and inhibits osteoblastic Smad activation through NF-kappaB. J. Bone Miner. Res..

[128] Gilbert L., He X., Farmer P., Rubin J., Drissi H., van Wijnen A.J., Lian J.B., Stein G.S., Nanes M.S. (2002). Expression of the osteoblast differentiation factor RUNX2 (Cbfa1/AML3/Pebp2alpha A) is inhibited by tumor necrosis factor-alpha. J. Biol. Chem..

[129] Ghali O., Chauveau C., Hardouin P., Broux O., Devedjian J.C. (2010). TNF-alpha’s effects on proliferation and apoptosis in human mesenchymal stem cells depend on RUNX2 expression. J. Bone Miner. Res..

[130] Gilbert L., He X., Farmer P., Boden S., Kozlowski M., Rubin J., Nanes M.S. (2000). Inhibition of osteoblast differentiation by tumor necrosis factor-alpha. Endocrinology.

[131] Tartaglia L.A., Pennica D., Goeddel D.V. (1993). Ligand passing: The 75-kDa tumor necrosis factor (TNF) receptor recruits TNF for signaling by the 55-kDa TNF receptor. J. Biol. Chem..

[132] Beltinger C.P., White P.S., Maris J.M., Sulman E.P., Jensen S.J., LePaslier D., Stallard B.J., Goeddel D.V., de Sauvage F.J., Brodeur G.M. (1996). Physical mapping and genomic structure of the human TNFR2 gene. Genomics.

[133] Brenner D., Blaser H., Mak T.W. (2015). Regulation of tumour necrosis factor signalling: Live or let die. Nat. Rev. Immunol..

[134] Grell M., Douni E., Wajant H., Lohden M., Clauss M., Maxeiner B., Georgopoulos S., Lesslauer W., Kollias G., Pfizenmaier K. (1995). The transmembrane form of tumor necrosis factor is the prime activating ligand of the 80 kDa tumor necrosis factor receptor. Cell.

[135] Zhang Y.W., Yasui N., Ito K., Huang G., Fujii M., Hanai J., Nogami H., Ochi T., Miyazono K., Ito Y. (2000). A RUNX2/PEBP2alpha A/CBFA1 mutation displaying impaired transactivation and Smad interaction in cleidocranial dysplasia. Proc. Natl. Acad. Sci. USA.

[136] Afzal F., Pratap J., Ito K., Ito Y., Stein J.L., van Wijnen A.J., Stein G.S., Lian J.B., Javed A. (2005). Smad function and intranuclear targeting share a Runx2 motif required for osteogenic lineage induction and BMP2 responsive transcription. J. Cell Physiol..

[137] Guo R., Yamashita M., Zhang Q., Zhou Q., Chen D., Reynolds D.G., Awad H.A., Yanoso L., Zhao L., Schwarz E.M. (2008). Ubiquitin ligase Smurf1 mediates tumor necrosis factor-induced systemic bone loss by promoting proteasomal degradation of bone morphogenetic signaling proteins. J. Biol. Chem..

[138] Kaneki H., Guo R., Chen D., Yao Z., Schwarz E.M., Zhang Y.E., Boyce B.F., Xing L. (2006). Tumor necrosis factor promotes Runx2 degradation through up-regulation of Smurf1 and Smurf2 in osteoblasts. J. Biol. Chem..

[139] Nakase T., Takaoka K., Masuhara K., Shimizu K., Yoshikawa H., Ochi T. (1997). Interleukin-1 beta enhances and tumor necrosis factor-alpha inhibits bone morphogenetic protein-2-induced alkaline phosphatase activity in MC3T3-E1 osteoblastic cells. Bone.

[140] Chen S., Guttridge D.C., Tang E., Shi S., Guan K., Wang C.Y. (2001). Suppression of tumor necrosis factor-mediated apoptosis by nuclear factor kappaB-independent bone morpho.ogenetic protein/Smad signaling. J. Biol. Chem..

[141] Abbas S., Zhang Y.H., Clohisy J.C., Abu-Amer Y. (2003). Tumor necrosis factor-alpha inhibits pre-osteoblast differentiation through its type-1 receptor. Cytokine.

[142] Gomathi K., Akshaya N., Srinaath N., Moorthi A., Selvamurugan N. (2020). Regulation of Runx2 by post-translational modifications in osteoblast differentiation. Life Sci..

[143] Jilka R.L., Weinstein R.S., Bellido T., Parfitt A.M., Manolagas S.C. (1998). Osteoblast programmed cell death (apoptosis): Modulation by growth factors and cytokines. J. Bone Miner. Res..

[144] Mogi M., Togari A. (2003). Activation of caspases is required for osteoblastic differentiation. J. Biol. Chem..

[145] Xin W., Wang X., Zhang W., Zhu H., Dong R., Zhang J. (2019). Tumor Necrosis Factor-alpha Inhibits Bone Marrow Stem Cell Differentiation into Osteoblasts by Downregulating microRNA-34a Expression. Ann. Clin. Lab. Sci..

[146] Hill P.A., Tumber A., Meikle M.C. (1997). Multiple extracellular signals promote osteoblast survival and apoptosis. Endocrinology.

[147] Tsuboi M., Kawakami A., Nakashima T., Matsuoka N., Urayama S., Kawabe Y., Fujiyama K., Kiriyama T., Aoyagi T., Maeda K. (1999). Tumor necrosis factor-alpha and interleukin-1beta increase the Fas-mediated apoptosis of human osteoblasts. J. Lab. Clin. Med..

[148] Nanes M.S. (2003). Tumor necrosis factor-alpha: Molecular and cellular mechanisms in skeletal pathology. Gene.

[149] Panagakos F.S., Kumar S. (1994). Modulation of proteases and their inhibitors in immortal human osteoblast-like cells by tumor necrosis factor-alpha in vitro. Inflammation.

[150] Kuroki T., Shingu M., Koshihara Y., Nobunaga M. (1994). Effects of cytokines on alkaline phosphatase and osteocalcin production, calcification and calcium release by human osteoblastic cells. Br. J. Rheumatol..

[151] Mayur N., Lewis S., Catherwood B.D., Nanes M.S. (1993). Tumor necrosis factor alpha decreases 1,25-dihydroxyvitamin D3 receptors in osteoblastic ROS 17/2.8 cells. J. Bone Miner. Res..

[152] Katz M.S., Gutierrez G.E., Mundy G.R., Hymer T.K., Caulfield M.P., McKee R.L. (1992). Tumor necrosis factor and interleukin 1 inhibit parathyroid hormone-responsive adenylate cyclase in clonal osteoblast-like cells by down-regulating parathyroid hormone receptors. J. Cell Physiol..

[153] Kose K.N., Xie J.F., Carnes D.L., Graves D.T. (1996). Pro-inflammatory cytokines downregulate platelet derived growth factor-alpha receptor gene expression in human osteoblastic cells. J. Cell Physiol..

[154] Tsutsumimoto T., Kawasaki S., Ebara S., Takaoka K. (1999). TNF-alpha and IL-1beta suppress N-cadherin expression in MC3T3-E1 cells. J. Bone Miner. Res..

[155] Nagy Z., Radeff J., Stern P.H. (2001). Stimulation of interleukin-6 promoter by parathyroid hormone, tumor necrosis factor alpha, and interleukin-1beta in UMR-106 osteoblastic cells is inhibited by protein kinase C antagonists. J. Bone Miner. Res..

[156] Panagakos F.S., Kumar S. (1995). Differentiation of human osteoblastic cells in culture: Modulation of proteases by extracellular matrix and tumor necrosis factor-alpha. Inflammation.

[157] Kurokouchi K., Kambe F., Yasukawa K., Izumi R., Ishiguro N., Iwata H., Seo H. (1998). TNF-alpha increases expression of IL-6 and ICAM-1 genes through activation of NF-kappaB in osteoblast-like ROS17/2.8 cells. J. Bone Miner. Res..

[158] Yao G.Q., Sun B.H., Insogna K.L., Weir E.C. (2000). Nuclear factor-kappaB p50 is required for tumor necrosis factor-alpha-induced colony-stimulating factor-1 gene expression in osteoblasts. Endocrinology.

[159] Isaacs S.D., Fan X., Fan D., Gewant H., Murphy T.C., Farmer P., Taylor W.R., Nanes M.S., Rubin J. (1999). Role of NFkappaB in the regulation of macrophage colony stimulating factor by tumor necrosis factor-alpha in ST2 bone stromal cells. J. Cell Physiol..

[160] Jupp O.J., McFarlane S.M., Anderson H.M., Littlejohn A.F., Mohamed A.A., MacKay R.H., Vandenabeele P., MacEwan D.J. (2001). Type II tumour necrosis factor-alpha receptor (TNFR2) activates c-Jun N-terminal kinase (JNK) but not mitogen-activated protein kinase (MAPK) or p38 MAPK pathways. Biochem. J..

[161] Kalb A., Bluethmann H., Moore M.W., Lesslauer W. (1996). Tumor necrosis factor receptors (Tnfr) in mouse fibroblasts deficient in Tnfr1 or Tnfr2 are signaling competent and activate the mitogen-activated protein kinase pathway with differential kinetics. J. Biol. Chem..

[162] Gerstenfeld L.C., Cho T.J., Kon T., Aizawa T., Cruceta J., Graves B.D., Einhorn T.A. (2001). Impaired intramembranous bone formation during bone repair in the absence of tumor necrosis factor-alpha signaling. Cells Tissues Organs.

[163] Hofbauer L., Lacey D., Dunstan C., Spelsberg T., Riggs B., Khosla S. (1999). Interleukin-1β and tumor necrosis factor-α, but not interleukin-6, stimulate osteoprotegerin ligand gene expression in human osteoblastic cells. Bone.

[164] Robling A.G., Bonewald L.F. (2020). The Osteocyte: New Insights. Annu. Rev. Physiol..

[165] Zhou M., Li S., Pathak J.L. (2019). Pro-inflammatory Cytokines and Osteocytes. Curr. Osteoporos. Rep..

[166] Jilka R.L., O’Brien C.A., Roberson P.K., Bonewald L.F., Weinstein R.S., Manolagas S.C. (2014). Dysapoptosis of osteoblasts and osteocytes increases cancellous bone formation but exaggerates cortical porosity with age. J. Bone Miner. Res..

[167] Plotkin L.I. (2014). Apoptotic osteocytes and the control of targeted bone resorption. Curr. Osteoporos. Rep..

[168] Aguirre J.I., Plotkin L.I., Stewart S.A., Weinstein R.S., Parfitt A.M., Manolagas S.C., Bellido T. (2006). Osteocyte apoptosis is induced by weightlessness in mice and precedes osteoclast recruitment and bone loss. J. Bone Miner. Res..

[169] Cabahug-Zuckerman P., Frikha-Benayed D., Majeska R.J., Tuthill A., Yakar S., Judex S., Schaffler M.B. (2016). Osteocyte Apoptosis Caused by Hindlimb Unloading is Required to Trigger Osteocyte RANKL Production and Subsequent Resorption of Cortical and Trabecular Bone in Mice Femurs. J. Bone Miner. Res..

[170] Kennedy O.D., Herman B.C., Laudier D.M., Majeska R.J., Sun H.B., Schaffler M.B. (2012). Activation of resorption in fatigue-loaded bone involves both apoptosis and active pro-osteoclastogenic signaling by distinct osteocyte populations. Bone.

[171] Kogianni G., Mann V., Ebetino F., Nuttall M., Nijweide P., Simpson H., Noble B. (2004). Fas/CD95 is associated with glucocorticoid-induced osteocyte apoptosis. Life Sci..

[172] Lirani-Galvao A.P., Chavassieux P., Portero-Muzy N., Bergamaschi C.T., Silva O.L., Carvalho A.B., Lazaretti-Castro M., Delmas P.D. (2009). Low-intensity electrical stimulation counteracts the effects of ovariectomy on bone tissue of rats: Effects on bone microarchitecture, viability of osteocytes, and nitric oxide expression. Calcif. Tissue Int..

[173] Metzger C.E., Narayanan A., Zawieja D.C., Bloomfield S.A. (2017). Inflammatory Bowel Disease in a Rodent Model Alters Osteocyte Protein Levels Controlling Bone Turnover. J. Bone Miner. Res..

[174] Narayanan S.A., Metzger C.E., Bloomfield S.A., Zawieja D.C. (2018). Inflammation-induced lymphatic architecture and bone turnover changes are ameliorated by irisin treatment in chronic inflammatory bowel disease. FASEB J..

[175] Morita M., Iwasaki R., Sato Y., Kobayashi T., Watanabe R., Oike T., Nakamura S., Keneko Y., Miyamoto K., Ishihara K. (2017). Elevation of pro-inflammatory cytokine levels following anti-resorptive drug treatment is required for osteonecrosis development in infectious osteomyelitis. Sci. Rep..

[176] Ahuja S.S., Zhao S., Bellido T., Plotkin L.I., Jimenez F., Bonewald L.F. (2003). CD40 ligand blocks apoptosis induced by tumor necrosis factor alpha, glucocorticoids, and etoposide in osteoblasts and the osteocyte-like cell line murine long bone osteocyte-Y4. Endocrinology.

[177] Plotkin L.I., Weinstein R.S., Parfitt A.M., Roberson P.K., Manolagas S.C., Bellido T. (1999). Prevention of osteocyte and osteoblast apoptosis by bisphosphonates and calcitonin. J. Clin. Investig..

[178] Bakker A.D., Silva V.C., Krishnan R., Bacabac R.G., Blaauboer M.E., Lin Y.C., Marcantonio R.A., Cirelli J.A., Klein-Nulend J. (2009). Tumor necrosis factor alpha and interleukin-1beta modulate calcium and nitric oxide signaling in mechanically stimulated osteocytes. Arthritis Rheum..

[179] Pathak J.L., Bakker A.D., Luyten F.P., Verschueren P., Lems W.F., Klein-Nulend J., Bravenboer N. (2016). Systemic Inflammation Affects Human Osteocyte-Specific Protein and Cytokine Expression. Calcif. Tissue Int..

[180] Kim J.H., Kim A.R., Choi Y.H., Jang S., Woo G.H., Cha J.H., Bak E.J., Yoo Y.J. (2017). Tumor necrosis factor-alpha antagonist diminishes osteocytic RANKL and sclerostin expression in diabetes rats with periodontitis. PLoS ONE.

[181] Cheung W.Y., Simmons C.A., You L. (2012). Osteocyte apoptosis regulates osteoclast precursor adhesion via osteocytic IL-6 secretion and endothelial ICAM-1 expression. Bone.

[182] Tatsumi S., Ishii K., Amizuka N., Li M., Kobayashi T., Kohno K., Ito M., Takeshita S., Ikeda K. (2007). Targeted ablation of osteocytes induces osteoporosis with defective mechanotransduction. Cell Metab..

[183] Noble B.S., Stevens H., Loveridge N., Reeve J. (1997). Identification of apoptotic changes in osteocytes in normal and pathological human bone. Bone.

[184] Kogianni G., Mann V., Noble B.S. (2008). Apoptotic bodies convey activity capable of initiating osteoclastogenesis and localized bone destruction. J. Bone Miner. Res..

[185] Baek K., Hwang H.R., Park H.J., Kwon A., Qadir A.S., Ko S.H., Woo K.M., Ryoo H.M., Kim G.S., Baek J.H. (2014). TNF-alpha upregulates sclerostin expression in obese mice fed a high-fat diet. J. Cell Physiol..

[186] Poole K.E., van Bezooijen R.L., Loveridge N., Hamersma H., Papapoulos S.E., Lowik C.W., Reeve J. (2005). Sclerostin is a delayed secreted product of osteocytes that inhibits bone formation. FASEB J..

[187] Kang J., Boonanantanasarn K., Baek K., Woo K.M., Ryoo H.M., Baek J.H., Kim G.S. (2015). Hyperglycemia increases the expression levels of sclerostin in a reactive oxygen species- and tumor necrosis factor-alpha-dependent manner. J. Periodontal Implant. Sci..

[188] Ito N., Wijenayaka A.R., Prideaux M., Kogawa M., Ormsby R.T., Evdokiou A., Bonewald L.F., Findlay D.M., Atkins G.J. (2015). Regulation of FGF23 expression in IDG-SW3 osteocytes and human bone by pro-inflammatory stimuli. Mol. Cell Endocrinol..

[189] Guo Y.C., Yuan Q. (2015). Fibroblast growth factor 23 and bone mineralisation. Int. J. Oral Sci..

[190] Metzger C.E., Gong S., Aceves M., Bloomfield S.A., Hook M.A. (2019). Osteocytes reflect a pro-inflammatory state following spinal cord injury in a rodent model. Bone.

[191] Uluckan O., Jimenez M., Karbach S., Jeschke A., Grana O., Keller J., Busse B., Croxford A.L., Finzel S., Koenders M. (2016). Chronic skin inflammation leads to bone loss by IL-17-mediated inhibition of Wnt signaling in osteoblasts. Sci. Transl. Med..

